# Supplementary material for: Factors Associated with Large Cup-to-Disc Ratio and Blindness in the Primary Open-Angle African American Glaucoma Genetics (POAAGG) Study
Source: Genes (Basel). 2023 Sep 16;14(9):1809. doi: 10.3390/genes14091809 (PMC10530848; doi:10.3390/genes14091809)
Supplement: Supplementary file 1 [file genes-14-01809-s001.zip › genes-2581420-supplementary.pdf]

Supplements

Supplemental Table S1

Univariate analysis of demographic and clinical characteristics of eyes with and without LCDR.

Characteristic	Not Extreme CDR (n=4165 eyes)	Extreme CDR (n=1440 eyes)	Total (n=5,605)	Odds Ratio (95% CI)	P-value
Age, mean (SD), years (OR per 10 yr increase)	69.5 (11.1)	70.9 (11.9)	69.9 (11.4)	1.11(1.04-1.20)	0.003
Age Group [%]					
<60	746 (18.4%)	255 (18.9%)	1001 (18.5%)	Ref	
[60,70]	1237 (30.5%)	338 (25.0%)	1575 (29.1%)	0.80(0.63-1.01)	
[70,80]	1282 (31.6%)	394 (29.1%)	1676 (31.0%)	0.90(0.72-1.13)	
>=80	791 (19.5%)	365 (27.0%)	1156 (21.4%)	1.35(1.06-1.71)	<0.001
Gender, n [%]					
Male	1664 (40.0%)	816 (56.7%)	2480 (44.2%)	Ref	
Female	2501 (60.0%)	624 (43.3%)	3125 (55.8%)	0.51(0.44-0.59)	<0.001
BMI, mean ± SD (OR per 1 unit increase)	30.1 (6.6)	28.5 (6.5)	29.7 (6.6)	0.96(0.95-0.97)	<0.001
Diabetes, n (%)	1717 (43.3%)	475 (35.4%)	2192 (41.3%)	0.72(0.61-0.84)	<0.001
Hypertension, n (%)	3144 (79.4%)	1039 (77.2%)	4183 (78.9%)	0.88(0.73-1.06)	0.17
Family history of glaucoma, n (%)	2098 (57.0%)	731 (59.4%)	2829 (57.6%)	1.11(0.94-1.30)	0.23
Previous glaucoma surgery, n (%)	819 (20.9%)	641 (48.2%)	1460 (27.8%)	3.52(2.99-4.15)	<0.001
Tobacco use, n (%)	2027 (53.3%)	732 (56.7%)	2759 (54.1%)	1.15(0.98-1.35)	0.09
Alcohol use, n (%)	1943 (51.2%)	626 (48.8%)	2569 (50.6%)	0.91(0.77-1.06)	0.22
Ancestry (q0), mean ± SD (OR per 0.1 units increase)	0.403 (0.302)	0.374 (0.299)	0.395 (0.302)	0.97(0.94-1.00)	0.03

Supplemental Table S2

Univariate analysis of optic disc characteristics of eyes with and without LCDR

Characteristic	Not Extreme CDR	Extreme CDR	Total	Odds Ratio (95% CI)	P-value
Disc shape, n (%), total					
Round	1068 (42.1%), 2539	390 (43.9%), 889	1458 (42.5%), 3428	Ref	
Oval	1461 (57.5%), 2539	496 (55.8%), 889	1957 (57.1%), 3428	0.93(0.78-1.10)	
Other	10 (0.4%), 2539	3 (0.3%), 889	13 (0.4%), 3428	0.82(0.22-3.09)	0.69
Shape of cup, n (%), total					
Conical	1066 (43.6%), 2445	282 (33.7%), 838	1348 (41.1%), 3283	Ref	
Cylindrical	1184 (48.4%), 2445	282 (33.7%), 838	1466 (44.7%), 3283	0.90(0.74-1.10)	<0.001

Bean Pot	183 (7.5%), 2445	272 (32.5%), 838	455 (13.9%), 3283	5.62(4.34-7.28)	
Other	12 (0.5%), 2445	2 (0.2%), 838	14 (0.4%), 3283	0.63(0.14-2.82)	
Beta peripapillary atrophy, n (%) total	422 (16.7%), 2520	191 (22.0%), 868	613 (18.1%), 3388	1.40(1.13-1.74)	0.002
Stereoscopically identified disc tilt, n (%) total	275 (10.9%), 2516	74 (8.5%), 870	349 (10.3%), 3386	0.76(0.56-1.02)	0.07
Disc hemorrhage, n (%) total	47 (1.9%), 2536	13 (1.5%), 877	60 (1.8%), 3413	0.80(0.43-1.48)	0.47
Arteriole narrowing, n (%) total	34 (1.3%), 2531	19 (2.2%), 874	53 (1.6%), 3405	1.63(0.91-2.91)	0.1
Venule narrowing, n (%) total	33 (1.3%), 2532	17 (1.9%), 875	50 (1.5%), 3407	1.50(0.83-2.70)	0.18
Baring of the lamina cribrosa, n (%) total	1660 (66.1%), 2513	690 (79.8%), 865	2350 (69.6%), 3378	2.03(1.65-2.48)	<0.001
Vessel bayonetting, n (%) total	701 (28.6%), 2452	459 (54.4%), 843	1160 (35.2%), 3295	2.99(2.52-3.54)	<0.001
Nasalization of the vessels, n (%) total	753 (29.8%), 2530	551 (63.2%), 872	1304 (38.3%), 3402	4.05(3.38-4.86)	<0.001
Cilioretinal vessels, n (%) total	566 (22.4%), 2527	169 (19.4%), 873	735 (21.6%), 3400	0.83(0.68-1.02)	0.08
Gray crescent, n (%) total	235 (9.3%), 2535	72 (8.2%), 880	307 (9.0%), 3415	0.87(0.64-1.19)	0.39
Pallor of disc, n (%) total	29 (1.1%), 2535	108 (12.3%), 878	137 (4.0%), 3413	12.12(7.72-19.03)	<0.001
Visible pores in lamina cribrosa, n (%) total	1414 (57.5%), 2458	654 (77.8%), 841	2068 (62.7%), 3299	2.58(2.10-3.17)	<0.001

Supplemental Table S3

Univariate analysis of phenotypic characteristics between eyes with and without LCDR.

Characteristic	Not Extreme CDR	Extreme CDR	Total	Odds Ratio (95% CI)	P-value
Central corneal thickness (µm), mean (SD), N	536.7 (39.2), 3843	527.4 (39.9), 1321	534.3 (39.6), 5164	1.01(1.00-1.01)	<0.001
Intra-ocular pressure (mmHg), mean (SD), N	23.6 (7.2), 4156	29.1 (11.4), 1438	25.0 (8.8), 5594	1.07(1.06-1.08)	<0.001
Mean deviation (dB), mean (SD), N	-4.1 (6.0), 3210	-14.7 (10.9), 1003	-6.6 (8.7), 4213	1.163(1.15-1.18)	<0.001
Pattern standard deviation (dB), mean (SD), N	5.2 (3.3), 3217	7.9 (3.7), 1004	5.9 (3.6), 4221	1.23(1.20-1.25)	<0.001
RNFL (µm), mean (SD), N	79.0 (15.3), 3311	63.7 (14.1), 959	75.6 (16.3), 4270	1.10(1.09-1.11)	<0.001

Visual acuity (corrected, logMAR), mean (SD), N	0.4 (0.5), 3677	1.1 (1.4), 1254	0.6 (0.9), 4931	2.67(2.34-3.06)	<0.001

Supplemental Table S4

Univariable analysis for comparison of demographic characteristics between non-blind eyes vs. blind eyes among LCDR eyes.

Characteristic	Not blind (n=877 eyes)	Blind (n=377 eyes)	Total	Odds Ratio (95% Confidence Interval)	P-value
Age, mean (SD), years	70.6 (12.1)	72.0 (11.6)	71.0 (11.9)	1.10(0.99-1.23)	0.08
Age group					
<60	160 (71.4%)	64 (28.6%)	224 (18.7%)	Ref	0.12
[60,70]	218 (74.4%)	75 (25.6%)	293 (24.4%)	1.31(0.88-1.95)	
[70,80]	240 (67.8%)	114 (32.2%)	354 (29.5%)	0.86(0.57-1.31)	
>=80	216 (65.7%)	113 (34.3%)	329 (27.4%)	1.19(0.80-1.76)	
Gender					
Male	483 (55.1%)	212 (56.2%)	695 (55.4%)	Ref	0.72
Female	394 (44.9%)	165 (43.8%)	559 (44.6%)	0.95(0.74-1.24)	
BMI, mean (SD)	28.8 (6.4)	28.3 (6.9)	28.6 (6.5)	1.0(1.0-1.0)	0.30
Diabetes	292 (35.4%)	130 (36.3%)	422 (35.6%)	1.04(0.79-1.37)	0.77
Hypertension	637 (76.9%)	284 (78.9%)	921 (77.5%)	1.12(0.81-1.55)	0.49
Family history of glaucoma	436 (58.0%)	208 (61.9%)	644 (59.2%)	1.18(0.89-1.55)	0.25
Previous glaucoma surgery	350 (42.6%)	206 (58.4%)	556 (47.3%)	1.89(1.44-2.48)	<0.001
Tobacco use	430 (53.0%)	217 (61.0%)	647 (55.4%)	1.38(1.06-1.81)	0.02
Alcohol use	397 (49.2%)	145 (40.8%)	542 (46.6%)	0.71(0.55-0.93)	0.01
Ancestry (q0), mean (SD)	0.377 (0.298)	0.306 (0.263)	0.357 (0.290)	0.91(0.87-0.96)	<0.001

Supplemental Table S5

Univariable analysis for comparison of optic disc characteristics between non-blind eyes vs. blind eyes among LCDR eyes.

Characteristic	Not blind (n=604)	Blind (n=235 eyes)	Total	Odds Ratio (95% Confidence Interval)	P-value
Disc shape, n (%)					
Round	263 (43.5%)	108 (46.0%)	371 (44.1%)	Ref	0.54
Oval	341 (56.5%)	127 (54.0%)	468 (55.8%)	0.91(0.67-1.24)	
Shape of cup, n (%):					
Conical	180 (31.1%)	85 (39.5%)	265 (33.3%)	Ref	0.03
Cylindrical	215 (37.2%)	59 (27.4%)	274 (34.6%)	0.58(0.39-0.86)	
Bean Pot	183 (31.7%)	71 (33.0%)	254 (32.0%)	0.82(0.56-1.21)	
Beta peripapillary atrophy, n (%)	119 (19.7%)	64 (29.2%)	183 (22.3%)	1.68(1.16-2.43)	0.006

Stereoscopically identified disc tilt, n (%)	45 (7.5%)	23 (10.2%)	68 (8.3%)	1.40(0.79-2.49)	0.25
Disc hemorrhage, n (%)	9 (1.5%)	4 (1.8%)	13 (1.6%)	1.19(0.36-3.88)	0.78
Arteriole narrowing, n (%)	17 (2.8%)	2 (0.9%)	19 (2.3%)	0.31(0.07-1.33)	0.11
Venule narrowing, n (%)	8 (1.3%)	9 (4.0%)	17 (2.0%)	3.07(1.17-8.06)	0.02
Baring of the lamina cribrosa, n (%)	483 (80.9%)	169 (75.8%)	652 (79.5%)	0.74(0.51-1.07)	0.11
Vessel bayonetting, n (%)	330 (56.5%)	105 (48.6%)	435 (54.4%)	0.73(0.53-1.00)	0.053
Nasalization of the vessels, n (%)	387 (64.4%)	135 (59.7%)	522 (63.1%)	0.82(0.60-1.13)	0.23
Cilio retinal vessels, n (%)	120 (19.9%)	35 (15.6%)	155 (18.7%)	0.74(0.49-1.13)	0.16
Gray crescent, n (%)	53 (8.7%)	12 (5.2%)	65 (7.8%)	0.58(0.28-1.17)	0.13
Pallor of disc, n (%)	62 (10.2%)	39 (17.2%)	101 (12.1%)	1.82(1.17-2.83)	0.008
Visible pores in lamina cribrosa, n (%)	465 (79.8%)	152 (71.0%)	617 (77.4%)	0.62(0.43-0.90)	0.01

Supplemental Table S6

Univariable analysis for comparison of phenotypic characteristics between non-blind eyes vs. blind eyes among LCDR eyes.

Characteristic	Normal	Impaired or worse	Total	Odds Ratio (95% Confidence Interval)	P-value
Central Corneal Thickness (µm), mean (SD), N	526.1 (38.1), 814	522.8 (38.8), 349	525.1 (38.3), 1163	1.002(0.999-1.006)	0.21
Intra-Ocular Pressure (mmHg), mean (SD), N	27.2 (9.8), 877	34.2 (12.7), 377	29.3 (11.2), 1254	1.06(1.04-1.07)	<0.001
Mean Deviation (dB), mean (SD), N	-17.2 (9.6), 686	-22.0 (9.5), 202	-18.3 (9.8), 888	1.06(1.03-1.08)	<0.001
Pattern Standard Deviation (dB), mean (SD), N	8.1 (3.7), 685	7.0 (3.6), 203	7.9 (3.7), 888	0.92(0.88-0.96)	<0.001
RNFL Thickness (µm), mean (SD), N	60.1 (11.4), 672	60.5 (12.5), 204	60.2 (11.7), 876	1.00(0.98-1.01)	0.66
